# Complex Open Metacarpophalangeal Joint Dislocation of the Index Finger in a Pediatric Patient: A Case Report

**DOI:** 10.7759/cureus.49919

**Published:** 2023-12-04

**Authors:** Muhammed Uslu, Mahsum Solmaz, Mustafa Fatih Dasci, Ozan Beytemur

**Affiliations:** 1 Orthopedics and Traumatology, Health Sciences University, Bagcılar Training and Research Hospital, Istanbul, TUR; 2 Orthopedics and Traumatology, Health Sciences University, Bagcilar Training and Research Hospital, Istanbul, TUR

**Keywords:** case report, metacarpophalangeal joint dislocation, pediatric trauma, index finger, open wound, open injury, complex trauma

## Abstract

Pediatric metacarpophalangeal (MCP) joint dislocations are rare. Open MCP injuries are rarer. There are different surgical approaches to its treatment, and each approach has advantages and disadvantages. Debridement and open reduction should be performed urgently in the treatment. In our study, we will present the treatment and follow-up of a 15-year-old patient with an open index finger MCP joint dislocation. In conclusion, open MCP dislocations adversely affect hand function when their treatment is delayed, and complications can be avoided if full anatomical reduction and soft tissue reconstruction are performed quickly.

## Introduction

Metacarpophalangeal (MCP) joint dislocations are infrequent injuries, occurring less frequently than other types of dislocations in the fingers [1]. The mechanism of injury is hyperextension, which typically occurs after a fall onto an outstretched hand. Open MCP dislocations are rarer and occur with high-energy trauma [2]. Due to their greater susceptibility to trauma and the lack of support from the adjacent deep transverse metacarpal ligaments, the index, little fingers, and thumb are often affected [1]. These dislocations can be divided into simple and complex dislocations. Simple dislocations of the MCP joint can be reduced through closed techniques, while complex dislocations that require surgical intervention cannot be reduced by closed maneuvers [3,4]. The anatomy of complex dislocations involves multiple structures, but the volar plate is the most important structure that typically interferes with reduction through interposition with the MCP joint [5]. Open reduction using a dorsal or volar approach has been described for irreducible complex dislocations. It has been argued that the risk of damage to neurovascular structures is less with the dorsal approach, whereas the volar approach is advantageous because it allows better visualization of the pathological anatomy and volar plate repair [5]. In this article, we present the case of a 15-year-old patient with an open complex MCP joint dislocation of the index finger by evaluating two different approaches.

## Case presentation

**Figure 1:**
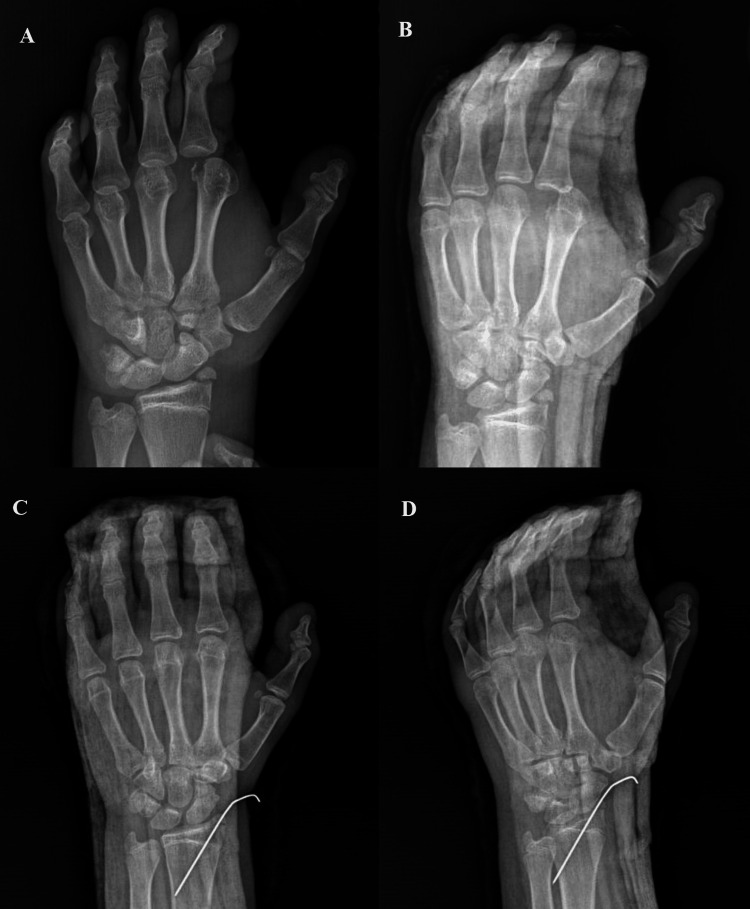
Preoperative (A, B) and postoperative (C, D) radiographic images show the dorsal dislocation of the left index metacarpophalangeal joint and concomitant radial styloid fracture

A 15-year-old male patient, with no prior medical history , fell to the ground with his hand open after an out-of-vehicle traffic accident and presented to our emergency department during the third hour of the trauma with an incision of approximately 7 cm in the palm. In the examination, it was seen that the dorsal part of the hand and the wrist were swollen, there was a dirty open wound of about 7 cm on the palm, and the second MCP joint remained in hyperextension and could not passively flex. Sensation and capillary refill time were normal over the finger. Plain radiographs confirmed dorsal dislocation of the right index MCP joint with enlarged joint space and concomitant radial styloid avulsion fracture (Figure 1).

Due to the dirty wound, it was classified as Gustilo grade III fracture, and tetanus toxoid and 1 gram of cefazolin prophylaxis were administered. The patient was sedated, and after copious irrigation of the wound with saline, an attempt was made by an orthopedic surgeon to reduce the dislocation in the emergency room, but it was unsuccessful. After the patient's parents were informed about the risks and benefits of surgical treatment, the patient accepted the surgery and was taken to the operating room four hours after the injury.

**Figure 2:**
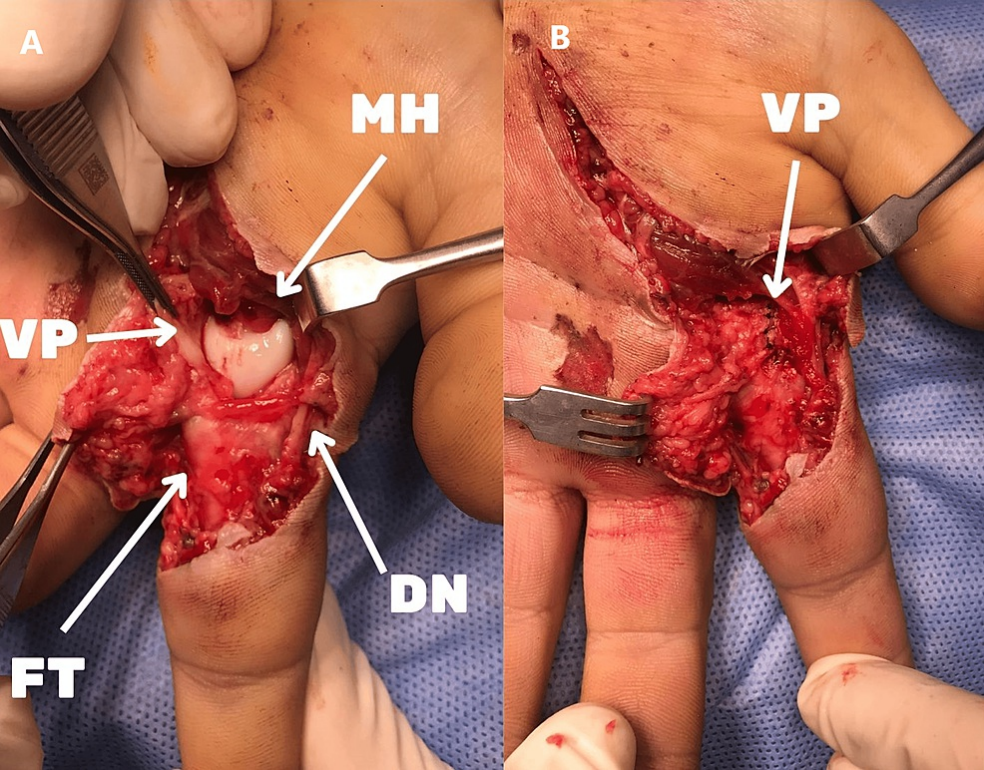
The relocated metacarpophalangeal joint and incised volar plate are visualized through the wound (A). The repaired volar plate is seen above the metacarpal head (B). MH: metacarpal head; VP: volar plate; DN: digital nerve; FT: flexor tendon

After the administration of general anesthesia in the operating room, the patient's existing incision line was thoroughly washed and debrided, and the metacarpal head was found to be volared and exposed. With blunt dissection, both digital arteries and nerves were found to be intact. It was observed that the first annular (A1) pulley was displaced, the flexor tendons were displaced radially, and the lumbrical muscle was displaced ulnarily. The volar plate was separated from the proximal attachment site. The reduction was attempted by flexing the wrist and finger and using a blunt elevator as a lever but failed. Thereupon, the reduction was achieved by making a longitudinal incision over the volar plate. The volar plate was repaired with a no. 4.0 polydioxanone (PD) suture and the incision line was repaired after copious washing (Figure 2).

Collateral ligaments were found to be intact on examination after reduction. Closed reduction and internal fixation were performed for the radial styloid fracture using one 1.5-mm Kirchner wire.

**Figure 3:**
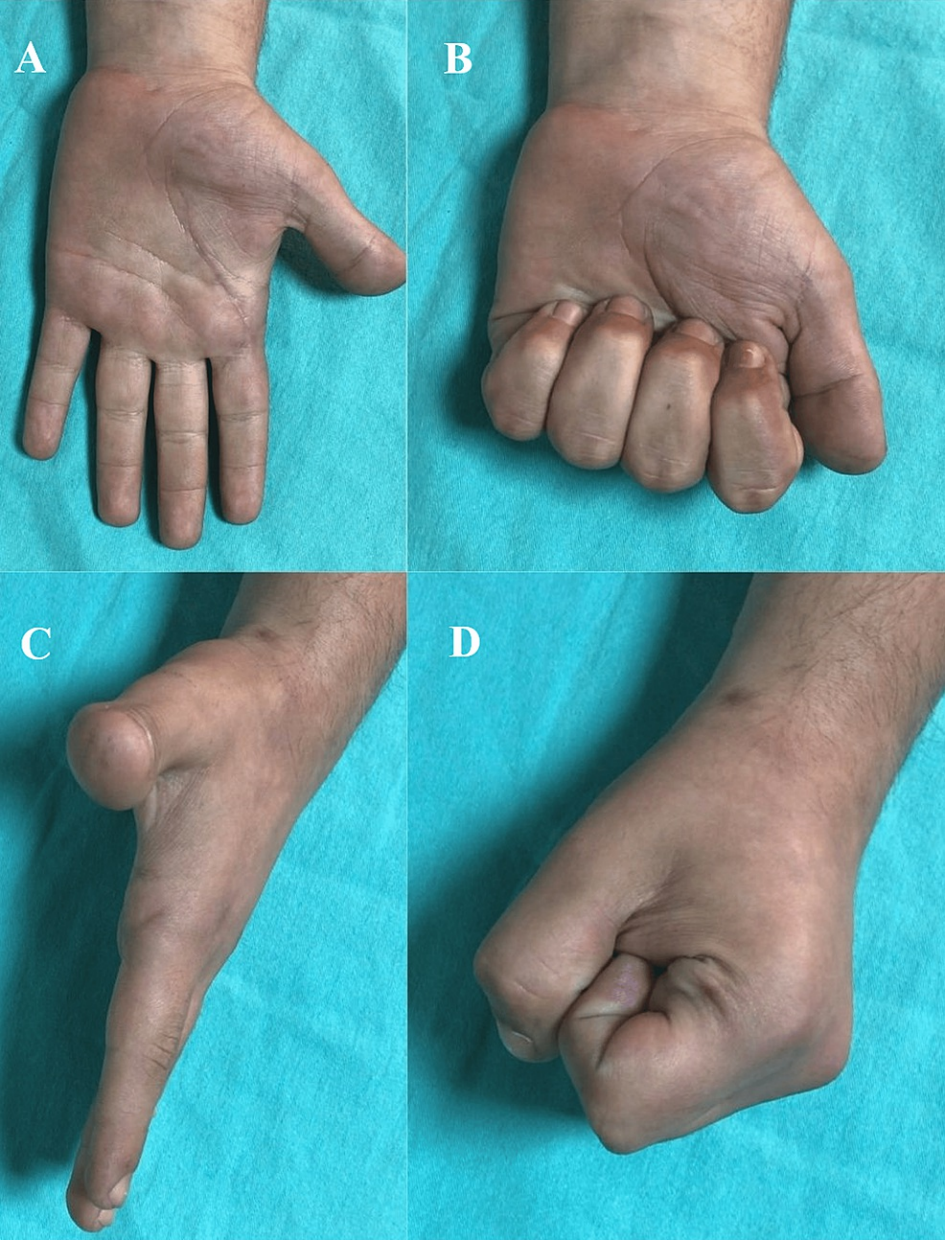
Functional result at week 12: flexion and extension of the metacarpophalangeal joint are normal.

A volar splint was applied to the MCP joint at 30 degrees of flexion, and the patient was discharged on the first postoperative day. At the outpatient clinic two weeks later, the splint was terminated and active movement was initiated by buddy taping. At six weeks postoperatively, the Kirchner wire in the radial styloid was removed. The patient had regained almost complete range of motion, and his radiographs showed that he had a concentric MCP joint. The patient did not have joint stiffness at the end of the one-year follow-up, and his imaging was unremarkable (Figure 3).

## Discussion

The MCP joint, a synovial joint, can perform many different movements. The complex anatomical structure of the joint allows circumduction, adduction, flexion, and extension. The MCP joint, like other synovial joints, is enclosed in a joint capsule that surrounds the joint. The volar plate on the palmar side of the MCP joint is connected to the proximal phalanx with a thick fibro-cartilaginous portion distally and to the metacarpus with a weak membranous portion proximally, supporting the joint capsule [4].

Dislocation of the MCP joint is a relatively rare injury. There are few case reports about this injury in the current literature. Dorsal MCP dislocations appear more frequently than volar dislocations. During such dislocations, the index finger, thumb, and little finger are frequently injured. Attention to technique is crucial when performing closed reduction on dorsal dislocations. It is imperative to adhere to the correct technique in order to achieve optimal results. To reduce simple dislocations, the wrist is flexed to loosen the flexor tendons, and pressure is applied distally and volarly to the dorsal base of the phalanx. If the reduction technique is not applied with care and traction and extension are applied, the entire volar plate may be pulled into the joint space and entrapped, leading to the development of a complex dislocation [6].

In complex dislocation of the MCP joint, the reduction is usually inhibited by the following structures: the volar plate, the A1 pulley, flexor tendons, lumbricals, and the abductor and flexor digiti quinti in the little finger. The periarticular tendons are pulled dorsally over the metacarpal head, while the volar plate is trapped dorsally by the dislocated proximal phalanx. In this way, a tendon loop forms around the neck of the metacarpal and is tightened further by traction. In index finger MCP dislocations, as in our patient, this tendon loop contains the lumbrical muscle on the radial side and flexor tendons on the ulnar side [2].

There are two different surgical approaches for complex dorsal MCP dislocations. The dorsal approach was first described by Farabeuf in 1876, and the volar approach by Kaplan in 1957 [5]. Both approaches have advantages and disadvantages. The volar approach allows for excellent visualization of trapped structures and their repair as needed. The index finger is exposed through an oblique volar incision. The radial neurovascular bundle always bends over the protruding index metacarpal head and lies just under the skin, as in our case. If the incision is not made carefully, it can be easily damaged. The dorsal approach allows the repair of existing osteochondral fractures and prevents damage to neurovascular structures. The dorsal approach provides excellent exposure for the evaluation of volar plaque trapped on the metacarpal head. A volar plate incision may be necessary to achieve reduction, but with a dorsal approach, the volar plate cannot be repaired and may cause instability [5].

In open fracture dislocations, as in our patient, the use of the incision where the existing incision line is may be preferred in order not to cause extra trauma to the patient. It is important to comply with the general open fracture principles and to wash with plenty of saline to prevent infection [2]. Dorsal dislocation and osteochondral fractures of the metacarpal head can be detected on lateral views. In our case, since the existing osteochondral fracture was not in the joint range, it was predicted that there would be no movement problem, and no intervention was made [7].

Repeated attempts at closed reduction, as well as traumatic or delayed open reductions in complicated dorsal MCP dislocations, can result in the development of degenerative arthritis and, in some cases, metacarpal head osteonecrosis [8]. It is vital to identify the location of the neurovascular bundle situated directly beneath the skin to prevent injury to it while surgically exposing the joint. It has been reported that patients with collateral ligament injuries after closed reduction do not develop stiffness even after three weeks of immobilization [9]. However, prolonged immobilization, delayed reduction, or concomitant extensive tissue injury may cause severe fibrosis, reducing the ultimate range of motion [10]. In children, because the physis is open, premature closure of the metacarpal head physis can be seen. Therefore, the family should be warned against this possibility [11].

## Conclusions

Although different incisions may be preferred for the treatment approach, it is important to use the incision at the site of injury for open injuries in complex MCP dislocations. It is important to follow the principles of open fracture treatment, to warn the patient and his or her family about fibrosis development and early closure of the physis, and to start early mobilization to prevent joint stiffness.
